# Transcriptome sequences spanning key developmental states as a resource for the study of the cestode *Schistocephalus solidus*, a threespine stickleback parasite

**DOI:** 10.1186/s13742-016-0128-3

**Published:** 2016-06-02

**Authors:** François Olivier Hébert, Stephan Grambauer, Iain Barber, Christian R. Landry, Nadia Aubin-Horth

**Affiliations:** Institut de Biologie Intégrative et des Systèmes (IBIS), Département de Biologie, Université Laval, Pavillon Charles-Eugène-Marchand, Québec, G1V 0A6, Canada; Department of Neuroscience, Psychology and Behaviour, Adrian Building, University of Leicester, University Road, Leicester, LE1 7RH, UK

**Keywords:** Transcriptome, RNA-seq, de novo assembly, *Schistocephalus solidus*, Parasite, Cestode, Flatworm, Threespine stickleback, *Gasterosteus aculeatus*

## Abstract

**Background:**

*Schistocephalus solidus* is a well-established model organism for studying the complex life cycle of cestodes and the mechanisms underlying host-parasite interactions. However, very few large-scale genetic resources for this species are available. We have sequenced and *de novo*-assembled the transcriptome of *S. solidus* using tissues from whole worms at three key developmental states - non-infective plerocercoid, infective plerocercoid and adult plerocercoid - to provide a resource for studying the evolution of complex life cycles and, more specifically, how parasites modulate their interactions with their hosts during development.

**Findings:**

The *de novo* transcriptome assembly reconstructed the coding sequence of 10,285 high-confidence unigenes from which 24,765 non-redundant transcripts were derived. 7,920 (77 %) of these unigenes were annotated with a protein name and 7,323 (71 %) were assigned at least one Gene Ontology term. Our raw transcriptome assembly (unfiltered transcripts) covers 92 % of the predicted transcriptome derived from the *S. solidus* draft genome assembly currently available on WormBase. It also provides new ecological information and orthology relationships to further annotate the current WormBase transcriptome and genome.

**Conclusion:**

This large-scale transcriptomic dataset provides a foundation for studies on how parasitic species with complex life cycles modulate their response to changes in biotic and abiotic conditions experienced inside their various hosts, which is a fundamental objective of parasitology. Furthermore, this resource will help in the validation of the *S solidus* gene features that have been predicted based on genomic sequence.

**Electronic supplementary material:**

The online version of this article (doi:10.1186/s13742-016-0128-3) contains supplementary material, which is available to authorized users.

## Data description

### Background

Parasites are increasingly recognized as critically important ecological agents that play a key role in nutrient cycling, influence inter-specific interactions and engineer the physicochemical properties of ecosystems [1]. Increased connectivity between trophic levels due to parasitic infections has been systematically investigated for more than 200 years. Peter Christian Abildgaard, a Danish veterinarian, was the first to identify a parasite being transmitted from one species to another via predation [2]. Abildgaard discovered the existence of complex parasite life cycles after observing that threespine sticklebacks (*Gasterosteus aculeatus*) and various fish-eating birds seemed to be infected by different forms of the same flatworm, named by Abildgaard as *Taenia gasterostei*. In a classic experiment, Abildgaard showed that ducks could acquire and maintain healthy *T. gasterostei* following the ingestion of infected threespine sticklebacks. This was the first demonstration of a complex life cycle, in which a parasite is transmitted from one host species to another [2]. After 155 years, in 1945, J.D. Smyth utilized *T. gasterostei*, by then named *Schistocephalus solidus*, as an experimental model. In an influential publication, Smyth described how *S. solidus* could be cultivated in vitro in the laboratory using specific experimental conditions to mimic the conditions within different hosts [3]. The infection of threespine stickleback by *S. solidus* has now become a model system in various research areas, including evolution, physiology, immunology, ecology, behavior (reviewed in [4]) and genomics of host parasite interactions, as fully sequenced genomes for *G. aculeatus* [5] and *S. solidus* [6, 7] are now available.

The life cycle of *S. solidus* can be simulated in vivo and in vitro using Smyth’s techniques [4, 8, 9]. In brief, eggs hatch in the water to release the coracidium larvae. Any species of cyclopoid copepod can eat the coracidium, after which the larva develops into the procercoid stage in the body cavity of its crustacean host. Threespine sticklebacks feed on infected copepods, allowing the procercoid to migrate into the coelom of the fish. The procercoid then undergoes a transformation to become an early, immature plerocercoid entering in a growth phase that will last 8–16 weeks. This growth period results in gains of up to 300 times its initial mass [10, 11]. Once the plerocercoid reaches a critical body mass (50 mg or more [12]) it is able to infect its final host, which is typically any species of piscivorous bird [13]. Trophic transmission of the competent (‘infective’) plerocercoid allows the parasite to complete its sexual maturation and reproduction, either by self- or cross-fertilization, in the digestive tract of the bird [14, 15]. The eggs are finally released in the water through the bird’s faeces.

Despite complex parasite life cycles being first described more than 200 years ago, the question of why and how some parasites evolved to acquire this complex strategy still remains elusive [16]. One approach to understanding the evolution of these strategies involves characterizing the molecular mechanisms that allow the parasite to transition from one stage to another as it transfers through several different hosts. The transcriptome of the parasite, consisting of all of the mRNA molecules that can be produced by the organism, represents a critical level of biological organization. It plays a key role in modulating the concentration of proteins at the interface of the molecular interactions between the parasite and its host [17, 18]. Changes in gene expression represent a major mechanism by which phenotypic traits can be ‘fine-tuned’ to achieve success in variable environments [19], including those experienced by parasites as they transit successive host species.

Understanding how parasites interact with their host environments and how they respond to changes in the biotic and abiotic conditions present at each stage is a fundamental objective of parasitology. Understanding these interactions at a molecular level requires the development of genetic resources. Here we present a comprehensive *de novo* transcriptome sequence that covers three key developmental life cycle stages of *S. solidus* that occur in vertebrate hosts*,* namely the non-infective plerocercoid, the infective plerocercoid and the adult. This experimental host-parasite system also represents a unique opportunity to collect valuable empirical data that will increase our knowledge of how parasites impact ecological and evolutionary processes, through effects on host behavior, sexual development and physiology [20–25]. Finally, this first large-scale transcriptomic dataset will help in the validation of the *S. solidus* gene features that have been predicted based on genome sequence.

### Specimen collection and laboratory infections

Parasites used in this study were obtained from experimentally-infected, laboratory-raised threespine sticklebacks at the University of Leicester (Leicester, England) according to previously described protocols [3, 26, 27]. Culturing and RNA extraction protocols are also available via the protocols.io repository [28]. Parasite eggs utilized in these experimental infections were previously produced from adult worms following the in vitro culture of plerocercoids [3] extracted from wild-caught threespine sticklebacks collected from Clatworthy Reservoir in Somerset, England (51°06’86”N, 3°35’39”W). Experimentally-infected fish were the F1 progeny of adult parents collected from the same lake as the parasite population, and from two other locations in the United Kingdom: Carsington Water in Derbyshire, England (53°06’05.09”N, 1°64’36.58”W) and Inverleith Pond in Edinburgh, Scotland (55°96’78.57”N, 3°21’67.21”W).

In brief, parasite eggs were placed in Petri dishes filled with tap water for two weeks and exposed to light to stimulate hatching. Hatched larvae were fed to laboratory-cultured copepods (*Cyclops strennus* Fischer) and three to four weeks later they were fed to the laboratory-raised threespine sticklebacks. Fish exposed to infected copepods were randomly selected and killed in a benzocaine solution (15 mM) between 10 and 17 weeks post-exposure. The timing of the sampling and subsequent dissection allowed both ‘non-infective’ plerocercoids (<50 mg) and ‘infective’ plerocercoids (>50 mg) to be recovered aseptically from the coelom of infected fish [12]. Non-infective worms were collected between 10 and 13 weeks post-infection, while infective worms were collected between 16 and 17 weeks post-infection. Additional infective plerocercoids extracted aseptically from naturally infected threespine sticklebacks caught from Clatworthy Reservoir were cultured in vitro to simulate the avian digestive tract environment [3] in order to obtain samples of the sexually mature adult worm life stage.

### RNA extraction and library preparation

All of the worms were washed carefully with UltraPure RNase free water (Ambion Inc., Austin, TX, USA) immediately after being extracted aseptically from the fish coelom (non-infective and infective plerocercoids) or collected from incubated test tubes (adults), then quickly cut with a scalpel into square pieces of five millimeters by five millimeters, placed into RNAlater (Ambion Inc., Austin, TX, USA) at 4 °C overnight, then transferred the next morning to −80 °C until RNA extraction. Total RNA was extracted from *S. solidus* worms following the method developed by Chomczynski & Sacchi [29], based on acid guanidinium thiocyanate-phenol-chloroform (Trizol® reagent, Invitrogen, Carlsbad, CA, USA).

Total RNA quality assessment using an Agilent 2100 Bioanalyzer® (Agilent, Santa Clara, CA, USA) revealed profiles similar to sub-optimal, or potentially degraded sample (see [30] for sample-specific profiles). However, this profile has been consistently observed across multiple independent cestode extractions (*unpublished data*) and has also been documented in other taxa from the *Platyhelminthes* phylum (classes *Trematoda*, *Tricladida*), *Nematoda* phylum (classes *Chromadorea*, *Adenophorea*) and other taxonomic groups including *Arthropoda* and therefore is not likely to indicate degradation [31]. These profiles are most likely the result of thermal conversion producing gap-deletion patterns in the 28S rRNA, ultimately leading to its fragmentation [31]. All of our RNA samples exhibited this same gap-deletion pattern.

Total RNA samples from 14 individual worms were used to produce individual TruSeq cDNA Illumina sequencing libraries (San Diego, CA, USA) according to the manufacturer’s protocol (see Table 1). Libraries were evenly and randomly distributed into three Illumina HiSeq 2000 lanes so that each lane contained samples from all three developmental stages. Sequencing was performed on the Illumina HiSeq 2000 system at Centre de Recherche du CHU de Québec (Québec, QC, Canada) to generate a total of 375 million 100 bp paired-end reads. RNA from one adult worm was used to prepare an additional Illumina TruSeq sequencing library used to perform preliminary optimization tests on assembly parameters (see Table 1, sample cltw.A.01). This library was sequenced on the MiSeq system at Plateforme d’analyses génomiques (Institut de Biologie Intégrative et des Systèmes, Université Laval, Québec) and yielded 19.4 million 300 bp paired-end sequences (deposited into the NCBI Sequence Read Archive (SRA) with accession number SAMN04296611 associated with BioProject PRJNA304161).

**Table 1:** *Schistocephalus solidus* specimens from three different developmental stages used to generate the *de novo* reference transcriptome^b^

Sample ID	Mass (mg)	Stage^a^	Library size (no. raw reads)	Platform	Ave. read quality (PHRED score)
ssol.cltw.NI.05	1.7	NI	95.7 M	HiSeq 2000	37
ssol.cltw. NI.08	1.5	NI	57.6 M	HiSeq 2000	37
ssol.cltw. NI.12	5.2	NI	29.5 M	HiSeq 2000	37
ssol.cltw. NI.13	1.8	NI	69.6 M	HiSeq 2000	37
ssol.**cltw. NI.14**	**3.4**	**NI**	**59.8 M**	HiSeq 2000	**37**
ssol.cltw. NI.22	7.7	NI	49.1 M	HiSeq 2000	37
ssol.cltw. NI.26	13.1	NI	53.7 M	HiSeq 2000	37
ssol.cltw. NI.63	102	I	37.5 M	HiSeq 2000	37
ssol.cltw. NI.67	90.1	I	42.3 M	HiSeq 2000	37
ssol.cltw. I.98–1	101.2	I	36.8 M	HiSeq 2000	37
**ssol.cltw.I.98–2**	**108.1**	**I**	**56.3 M**	HiSeq 2000	**37**
*ssol.cltw.A.01*	*164.5*	*A*	*38.8 M*	*MiSeq*	*38*
ssol.cltw.A.03	321	A	61.2 M	HiSeq 2000	37
**ssol.cltw.A.07**	**329.4**	**A**	**50.2 M**	HiSeq 2000	**37**
ssol.cltw.A.12	356	A	51.7 M	HiSeq 2000	37

^a^ *NI* non-infective, *I* infective, *A* adult

^b^Worms in bold were selected as the three representative samples to be used to perform the initial raw *de novo* assembly. All 14 HiSeq libraries were then used to eliminate redundancy in the final dataset and increase assembly quality, while sample cltw.a-01 (in italic) was used to perform preliminary optimization tests on assembly parameters

### Transcriptome assembly

**Fig. 1:**
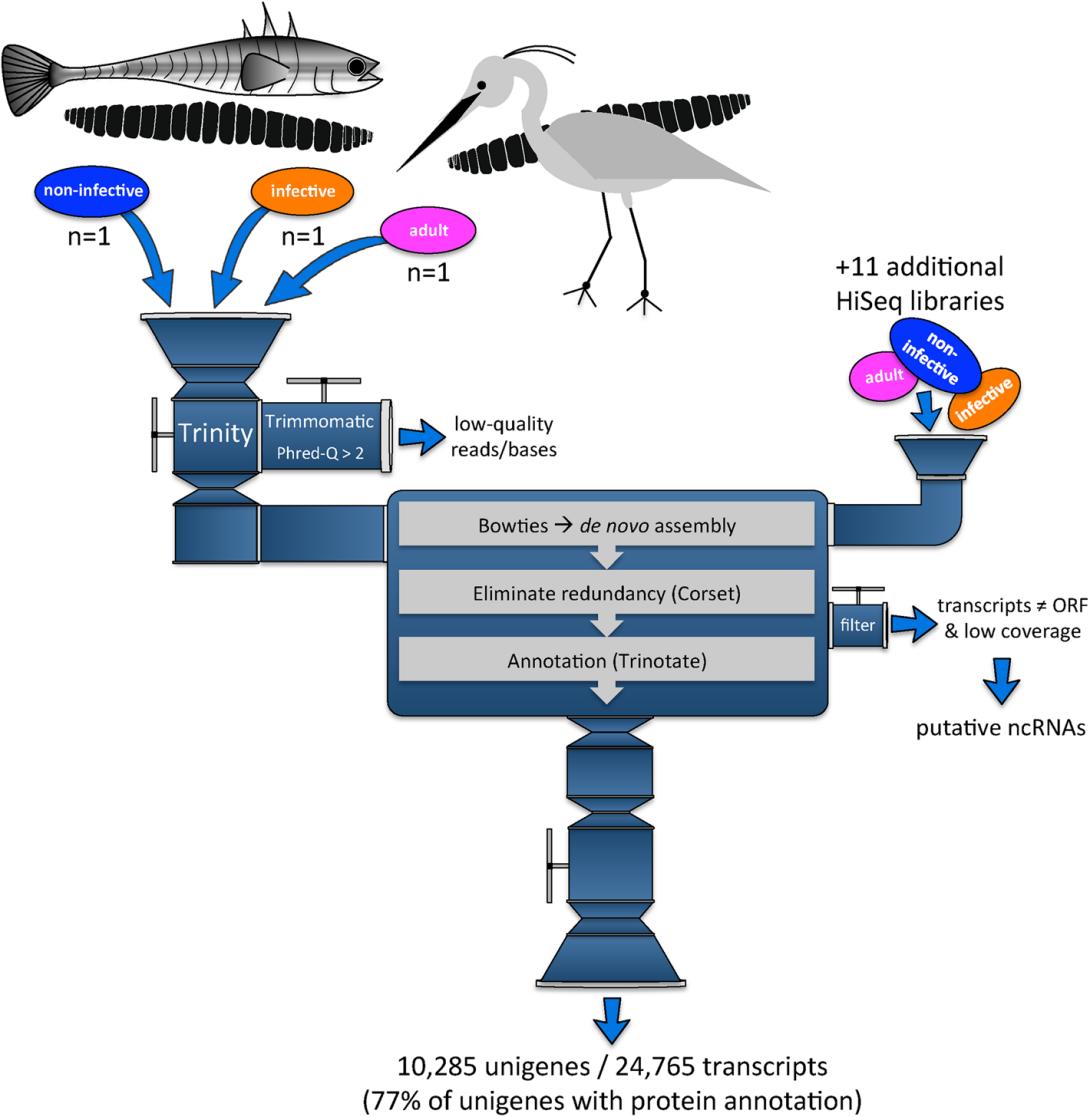
*De novo* assembly method used in the construction of a reference transcriptome for *Schistocephalus solidus*. Sequencing libraries from three developmental stages of *S. solidus*, non-infective plerocercoid, infective plerocercoid and adult, were trimmed (PHRED > 2, read length > 60 nucl.), concatenated and assembled *de novo* (1 library per stage). Next, the three libraries initially used to produce the *de novo* assembly, in addition to 11 libraries spanning the same three developmental stages (non-infective plerocercoid = 6 libraries, infective plerocercoid = 3 libraries, adult = 2 libraries) were aligned back to the *de novo* assembly. corset was used on the resulting alignment to eliminate redundancy by creating clusters of similar sequences, called ‘unigenes’. Transcripts were finally annotated through the Trinotate pipeline and transcripts poorly supported by protein-coding evidence were discarded, along with transcripts showing a low average coverage, i.e. CPM < 10 in 50 % of the samples in one group. The final transcriptome contains 24,765 transcripts accounting for 10,285 unigenes, of which 77 % could be annotated

Three worm libraries of similar size, one from each life stage were used for the initial *de novo* assembly (Table 1, *in bold*). Only one individual per life stage was used to obtain an initial set of raw *de novo* transcripts in order to i) minimize redundancy in assembled contigs due to allele splitting, ii) obtain the best possible balance between true transcript detection and false positives, and iii) obtain the maximum diversity of transcripts spanning all three life stages, i.e. characterize a maximum number of stage-specific genes [32–34]. In brief, reads from these pre-defined “representative worms” were combined for the initial assembly, then all 14 HiSeq libraries were used to reduce the final assembly and reduce contig redundancy as much as possible. The complete assembly pipeline is summarized in Fig. 1 and is available for download on Github [35].

Using Trimmomatic v0.33 [36], sequencing adaptors were removed from raw reads, reads were quality trimmed and then selected for minimum length (reads ≥ 60 bp were retained). As the primary goal of this study was to perform a *de novo* assembly, a less-stringent trimming threshold was selected (Phred score = 2) as suggested for increased *de novo* assembly quality [37]. Trimmed libraries for the three representative samples were then concatenated and used as the input for a *de novo* assembly through the Trinity pipeline v.2.0.6 with default parameters and a minimum contig length of 150 nucleotides [38]. Next, trimmed sequencing reads for the 14 HiSeq libraries (deposited into the NCBI SRA under accession number SRP066813, associated with BioProject PRJNA304161) were aligned with Bowtie 2 v.2.1.0 [39] against the *de novo* assembly, allowing multi-mapping for each read. Corset v.1.04 [40] was then utilized to cluster transcripts into unigenes based on sequence similarity and read counts (total of 14 sorted BAM files, i.e. one per individual library). Open reading frames (ORFs) were predicted for all transcripts with Transdecoder v.2.0.1 [38]. The raw transcriptome was finally filtered to discard transcripts that were poorly supported by protein-coding evidence (ORF length < 10 amino acids) and with low read counts (transcripts with CPM > 15 in at least three samples in one of the three developmental stages were kept). Considering the increasing evidence for key biological functions performed by non-coding RNAs [41], transcripts that did not contain ORFs that met our criteria were considered as “potentially non-coding” for further analysis and are provided as supplementary resource associated with this publication [30]. Results of the assembly and filtering steps can be found in Table 2.

**Table 2:** *De novo* assembly and annotations metrics for the transcriptome of the cestode *Schistocephalus solidus*

*De novo* assembly	
Assembled bases	195 089 904
Assembled transcripts	293 731
Unigenes – Unfiltered	115 318
Unigenes – Expression filter	12 291
Unigenes – Expression & ORF filters (transcripts)	10 285 (24 765)
Average transcripts per filtered unigene	2.41
Sum of filtered transcripts (Mbp)	367.83
Average length (bp) – Filtered transcripts (min - max : median)	2 684 (174–25 376 : 2168)
Annotation	
Unigenes with protein name	7 920 (77 %)
Unigenes with Gene Ontology (% of unigenes)	7 323 (71 %)
Proteins with complete ORF (% of unigenes)	20 335 (82 %)
Unigenes/transcripts with KEGG ID (% of unigenes)	4 270/7 798 (35 %)

### Annotation

Annotation was performed on predicted protein amino acid sequences using Trinotate v.2.0.2 [38] to assign a protein name and GO terms to each transcript. Predicted proteins were analyzed by several methods for functional annotation, starting with a sequence homology search on UniProtKB/Swissprot (downloaded 11 May 2015). Protein sequences were then mined for functional domains through HMMER v3.1 [42] and Pfam v.28.0 [43]. Signal peptides and transmembrane domains were assigned to coding sequences according to hidden Markov model-based prediction algorithms implemented in SignalP v.4.1 [44] and tmHMM v.2.0 [45], respectively. Transcripts were finally compared to curated annotation databases including EMBL Uniprot [46], KEGG [47], eggNOG v.3.0 [48] and GO pathways [49]. The steps and scripts built to implement this annotation pipeline are available on Github [50]. Transvestigator [51] was finally used to prepare the data for submission to NCBI Transcriptome Shotgun Assembly (TSA), by confirming that ORFs were on the positive strand and that each transcript was associated with at least one ORF. Annotation information based on the results obtained with Trinotate was also included in the TSA submission (accessible through GigaDB accession publication associated with this publication, see [30]).

### Comparison with gene-prediction models

A predicted transcriptome for *S. solidus* is currently available on WormBase v1.5.4 [6]. These gene predictions were generated by the Parasite Genomics group at the Wellcome Trust Sanger Institute from the genome by a combination of programs such as MAKER [52] and Augustus [53], as well as protein sequence homology searches against the taxonomically nearest reference helminth genome. Although gene prediction models can generate informative data when working with genome sequences, an essential task in characterizing gene features in a newly sequenced genome is to confirm and validate predicted coding sequences with empirical mRNA data [54]. The *de novo* transcriptome generated here was thus compared to the predicted version of the transcriptome and the complete genome from WormBase using two complementary approaches. First, a reciprocal best-hit analysis was performed, and second, our mRNA sequencing reads were aligned to the reference genome. We expected that only a partial representation of the predicted coding sequences on WormBase would be observed in the data presented here. This prediction stems from the fact that our *de novo* transcriptome was assembled using mRNA sequences for parasite stages in the fish and the bird hosts only. The developing embryo, free-swimming larval and procercoid stages were not considered in this assembly.

Results of a standard BLAST approach showed that 7,399 (72 %) of *de novo* unigenes (i.e. unigenes with valid ORF(s) and evidence of expression) give significant blast results (*e* < 1e-10, minimum 50 % overlap) when compared to the WormBase predicted transcriptome. Using reciprocal best hit BLAST [55] reduces the number of *de novo* unigenes with significant matches on the predicted transcriptome to 5,176 (50 %). Using the genome as a target, 9,877 (96 %) of our *de novo* unigenes return a significant *blastn* match (*e-value* < 1e-10, minimum 50 % overlap) on the WormBase genome with an average and a median sequence similarity of 92 % (range = 62–100 %, mode = 100 %, see Additional file 1). Recent work on the landscape genetics of *S. solidus* in Alaska (USA) revealed significant genetic differences among populations from several lakes along a gradient of isolation by distance [56]. The strong population structure and low admixture levels found in these lakes are indicative of low migration rates among populations. This could help explain why we obtain a median similarity of 92 % and not higher when comparing populations from the UK (*de novo* transcriptome generated in this study) with populations from Germany (WormBase genome).

As a complement to the BLAST approach, reads used to construct the *de novo* assembly were also aligned on the genome and predicted transcriptome using the bwa-mem algorithm [57] with default parameters. Mapping results indicate that 84 % of the reads successfully align on the genome (MAPQ ≥ 15), and 51 % of the reads successfully align on the predicted transcriptome. In total, 15,840 (78 %) predicted transcripts show some evidence of expression. The partial correspondence between the *de novo* assembly and the predicted transcriptome, as shown by the two complementary approaches, confirms our initial prediction that only a subset of all the possible genes in *S. solidus* would be represented in the stages assessed in this *de novo* transcriptome. On the other hand, 21 % of the *de novo* assembled transcripts exhibiting a valid ORF and evidence of expression across several individual worms were not represented in the predicted transcriptome. These transcripts were however detected in the genome, highlighting the importance of using RNA-seq data to further improve genome assemblies and annotations based on gene prediction models [58]. As only 84 % of the reads from the *de novo* transcriptome align on the genome (and not 100 %) may indicate i) regions missing from the current reference genome, ii) reads not mapping properly due to low complexity sequences, or iii) that polymorphisms between the individuals prevents mapping. Globally, these results call for a collaborative strategy taking advantage of multiple sources of information of a genomic and transcriptomic nature, towards an integrated and complete characterization of the genome structure of *S. solidus*.

An un-gapped *blastn* analysis of our raw transcriptome (unfiltered transcripts) against the WormBase predicted transcriptome revealed that it covers 92 % (18,608 transcripts) of the predicted transcripts, suggesting that some of the transcripts discarded in our pipeline based on protein-coding evidence and expression levels might in fact be true transcripts encoded in the genome. We consider these transcripts as ‘putative ncRNAs’. They are available in the public repository associated with this publication [30].

### Novel resource for phylogenomic analyses

**Fig. 2:**
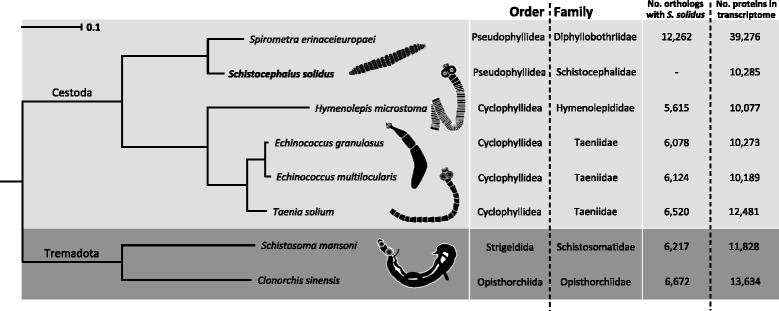
Phylogenetic relationships between *Schistocephalus solidus* (Schistocephalidae) and seven other parasitic worm species. These other species include five cestodes from the Cyclophyllidea and Pseudophyllidea orders and two trematodes (outgroups). More specifically, species from the cestode phylum include *Hymenolepis microstoma* (rodent tapeworm), *Taenia solium* (pork tapeworm), *Echinococcus multilocularis* (fox tapeworm), *Echinococcus granulosus* (dog tapeworm) and *Spirometra erinaceieuropaei* (responsible for the sparganosis infection), while the trematode outgroups are represented by *Schistosoma mansoni* (responsible for diseases such as schistosomiatis) and *Clonorchis sinensis* (Chinese liver fluke). Bootstrap values were all 100. Total number of single-copy orthologs used to produce the phylogenetic tree = 4 520 (distributed in 565 core orthologous groups). The number of orthologs shared with *S. solidus* is defined as the number of amino acid sequences in a given species that are part of an orthologous group identified by orthoMCL that also contains sequences from *S. solidus*

Establishing the evolutionary relationships among parasitic species represents a fundamental step towards understanding how parasitism evolved and how complex life cycles were acquired by certain taxa [59–62]. To date, very few genomes from parasitic worm species belonging to the *Pseudophyllidea* order (Phylum: *Platyhelminthes*, Class: *Cestoda*) have been fully sequenced [63] and currently there are no empirical transcriptomic resources available for any species belonging to the *Schistocephalidae* family, apart from the work on *Schistocephalus solidus* presented here. This new resource gives us an opportunity to fill this knowledge gap about evolutionary relationships. We assessed sequence homology between our *de novo* transcriptome and transcriptomes from seven other parasitic worm species by using OrthoMCL v.2.0.9 [64] according to the protocol developed by Fischer et al. [65]. Specifically, we included coding sequences from Wormbase ParaSite [66] for *Hymenolepis microstoma* (cestode, *Hymenolepiditae* family), *Taenia solium*, *Echinococcus granulosus*, *Echinococcus multilocularis* (cestodes, *Taenidae* family, described in [67]), *Spirometra erinaceieuropaei* (cestode, *Diphyllobothriidae* family, described in [63]) in addition to *Schistosoma mansonii* (trematode, *Schistosomatidae* family) and *Clonorchis sinensis* (tremadote, *Opisthorchiidae*) as outgroups in our analysis. Phylogenetic relationships among these eight species were built using a set of 565 groups of orthologous genes identified with OrthoMCL, each containing one single-copy gene per worm species (“single-copy orthologs”). Per species, single-copy orthologs were concatenated and aligned against one another using MAFFT v.7.245 [68]. Well-aligned regions were extracted using Gblocks 0.91b [69], which resulted in 80,865 aligned amino acid positions in 1,607 selected blocks. This final alignment was used to construct a phylogenetic tree with RAxML v.8.2.0 [70], following a gamma model rate of heterogeneity, combined with a WAG substitution matrix and a maximum likelihood search of 100 bootstraps. The resulting tree presented in Fig. 2 was visualized with Dendroscope v.3.4.4 [71]. Orthologous amino acid sequences for each species are provided in the GigaDB accession associated with this publication, as well as a full list of all potential orthologs between the species. In total, 8,329 unigenes (80 %) were identified as putative orthologs by OrthoMCL, meaning they show a sufficiently high sequence similarity to be aligned with at least one of the worm species included in the analysis. The results also suggest that 1,637 of the filtered unigenes (16 %) could potentially be specific to *S. solidus* since OrthoMCL identified them as singletons (no orthologous hit to any species included in the analysis). These specific unigenes may hold important species-specific information about this species and will be important to explore further. Of this subset, 43 were annotated with putative protein names, and 57 % remained as unknowns.

In conclusion, despite its position as a historical model system for the development of laboratory techniques now widely used in parasitology [72], *S. solidus* still remains an understudied species in terms of genomics. With novel resources such as the *de novo* transcriptome described here, *S. solidus* may additionally be a model for the study of conserved functions among parasitic worms, as well as offering species-specific genomic traits, among which may provide insight on key components of the complex life cycle of this model parasite.

## Availability and requirements

- Project name: Corset & Trinotate pipelines
- Project home page: https://github.com/fohebert/corset_pipeline & https://github.com/fohebert/Trinotate_pipeline
- Operating system(s): Unix.
- Programming language: Bash.
- Other requirements: TRIMMOMATIC, Trinity, Bowtie, CORSET, Samtools, limma-voom.
- License: GNU GPL v3
- None.

## Availability of supporting data

The raw datasets supporting the results of this article are available in the GigaDB repository associated with this publication [30]. All the sequencing data are available and associated with the NCBI BioProject PRJNA304161. Culturing and RNA extraction protocols are also available via the protocols.io repository [28].

### Ethical statement

Fish were captured under U.K. Environment Agency permit and with the permission of the landowner. All experiments were undertaken under a U.K. Home Office license (PPL80/2327), in accordance with local and national regulations, and in line with ABS/ASAB guidelines for the ethical treatment of animals in behavioral research.

## Additional file

Additional file 1: Sequence comparison between the genome and the *de novo* transcriptome. Description: Distribution of sequence similarities between the reference genome from WormBase and the *de novo* transcriptome. (PDF 450 kb)

## Abbreviations

CPM: counts per million
GO: Gene Ontology
ncRNA: non-coding RiboNucleic Acid
ORF: open reading frame
RNA: Ribonucleic acid
SRA: Sequence Read Archive
TSA: Transcriptome Shotgun Assembly
