# Supplementary figures and images for: Transcriptome sequences spanning key developmental states as a resource for the study of the cestode Schistocephalus solidus, a threespine stickleback parasite
Source: Gigascience. 2016 Jun 2;5:24. doi: 10.1186/s13742-016-0128-3 (PMC4891850; doi:10.1186/s13742-016-0128-3)

Sequence similarity comparison between the  
*de novo* transcriptome and the genome

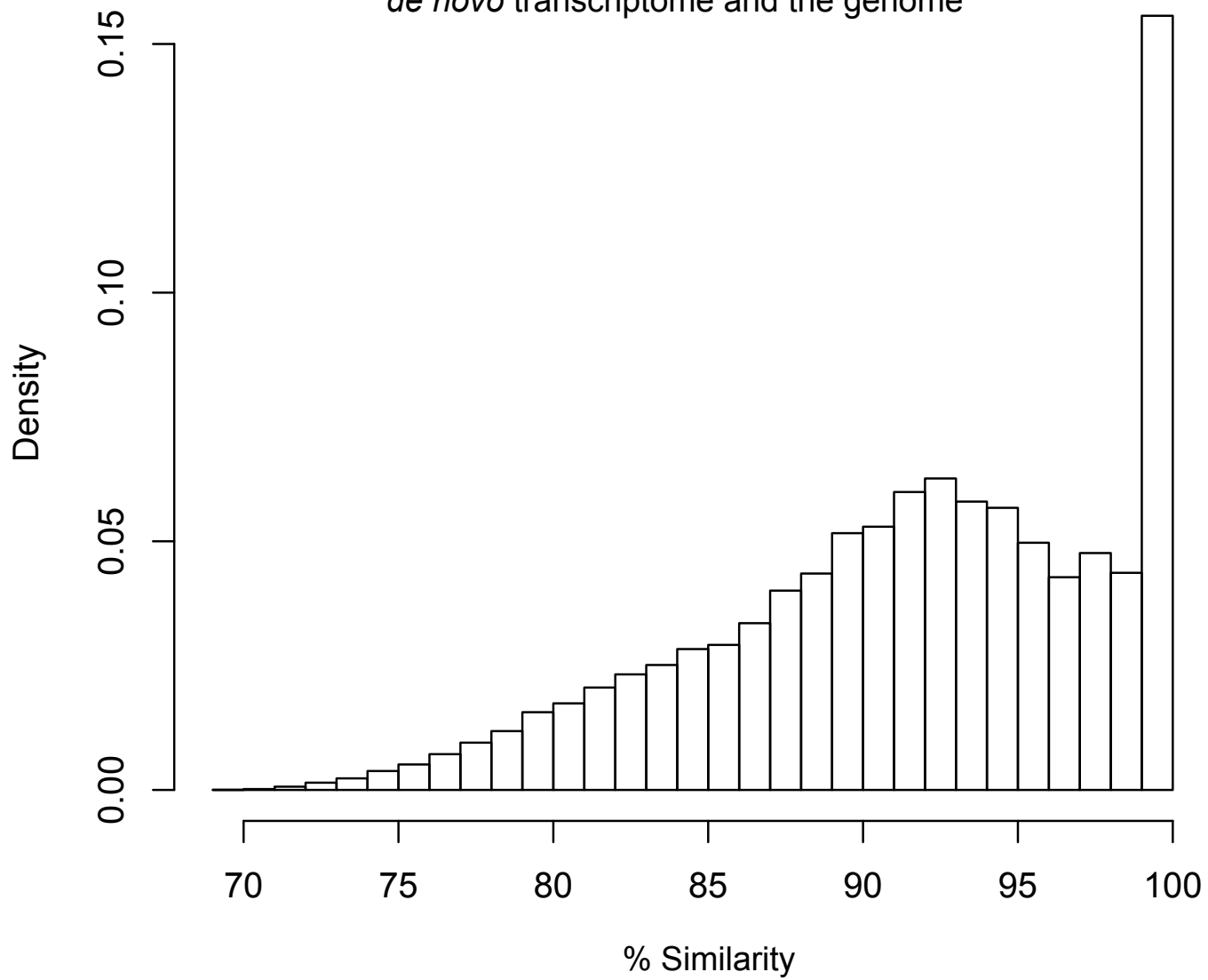

Supplement: Additional file 1: — Sequence comparison between the genome and the de novo transcriptome. Description: Distribution of sequence similarities between the reference genome from WormBase and the de novo transcriptome. (PDF 450 kb) [file 13742_2016_128_MOESM1_ESM.pdf]
